# Ferroelectricity and Self-Polarization in Ultrathin Relaxor Ferroelectric Films

**DOI:** 10.1038/srep19965

**Published:** 2016-01-28

**Authors:** Peixian Miao, Yonggang Zhao, Nengneng Luo, Diyang Zhao, Aitian Chen, Zhong Sun, Meiqi Guo, Meihong Zhu, Huiyun Zhang, Qiang Li

**Affiliations:** 1 Department of Physics and State Key Laboratory of Low-Dimensional Quantum Physics, Tsinghua University, Beijing 100084, China; 2 Collaborative Innovation Center of Quantum Matter, Beijing 100084, China; 3 Department of Chemistry, Tsinghua University, Beijing 100084, China

## Abstract

We report ferroelectricity and self-polarization in the (001) oriented ultrathin relaxor ferroelectric PMN-PT films grown on Nb-SrTiO_3_, SrRuO_3_ and La_0.7_Sr_0.3_MnO_3_, respectively. Resistance-voltage measurements and AC impedance analysis suggest that at high temperatures Schottky depletion width in a 4 nm thick PMN-PT film deposited on Nb-SrTiO_3_ is smaller than the film thickness. We propose that Schottky interfacial dipoles make the dipoles of the nanometer-sized polar nanoregions (PNRs) in PMN-PT films grown on Nb-SrTiO_3_ point downward at high temperatures and lead to the self-polarization at room temperature with the assistance of in-plane compressive strain. This work sheds light on the understanding of epitaxial strain effects on relaxor ferroelectric films and self-polarization mechanism.

Relaxor ferroelectrics have attracted much attention due to their intriguing properties and advantages for applications^1^,^2^,^3^,^4^,^5^,^6^,^7^,^8^,^9^,^10^,^11^. One of the most remarkable properties of relaxor ferroelectrics is the huge dielectric response with strong frequency dispersion within a large temperature range and the dielectric response shows a maximum at a certain temperature (T_m_). This response is related to the PNRs which start to form around the Burns temperature (*T*_B_)^1^,^7^, and the PNRs are induced by local random electric fields originating from charged compositional fluctuations^2^,^6^. The relaxor ferroelectrics are superior to the normal ferroelectrics in many aspects, such as huge electromechanical response, giant dielectric permittivity, strong pyroelectric and other effects^12^, which make relaxor ferroelectrics important for applications. However, the wide use of relaxor ferroelectric films is hindered by the lack of knowledge of epitaxial strain effects on relaxor ferroelectric properties^12^. This is in contrast to the case for epitaxial strain effects on the normal ferroelectric films, which have a lot of work^13^,^14^,^15^,^16^. It has been shown that epitaxial in-plane compressive strain favors ferroelectricity^13^,^14^,^15^,^16^ and even ultrathin films show ferroelectricity. For epitaxial relaxor ferroelectric films, theoretical calculations predicted that the growing in-plane strain can induce rotation of PNRs, or drive elongation of their dipoles, and even increase in the average size of the PNRs^17^. It was also predicted that the dipoles of PNRs point up and down under large in-plane compressive strain and in-plane compressive strain increases T_m_^17^. Until now these ideas have not been reported in thin relaxor ferroelectric films experimentally, especially in the ultrathin relaxor ferroelectric films. Tyunina *et al*. studied the epitaxial strain effects on relaxor ferroelectric films, including PbSc_0.5_Nb_0.5_O_3_ and PbMg_1/3_Nb_2/3_O_3_^12^. They found that in-plane compressive strain decreases T_m_, in contradiction to the case of normal ferroelectrics for which in-plane compressive strain favors ferroelectricity. In contrast, Nagarajan *et al*. studied the effect of strain on T_m_ of 0.9PMN-0.1PT relaxor ferroelectric thin films^18^,^19^. They tuned the strain in two ways. One is by growing 0.9PMN-0.1PT thin films with different thicknesses on LaAlO_3_ substrates^18^. Another one is by growing 0.9PMN-0.1PT thin films with a thickness of 100 nm on different substrates with different lattice mismatches^19^. In both cases, they found that in-plane compressive strain increases T_m_ while in-plane tensile strain decreases T_m_. So further work is needed to clarify the effect of strain on T_m_ and ferroelectricity of relaxor ferroelectric thin films. Moreover, the thinnest films used in their work are 25 nm (Ref. 12) and 100 nm (Refs 18 and 19), which are not ultrathin. Therefore, experimental exploration of epitaxial strain effects on relaxor ferroelectric films, especially the ultrathin films, is highly desired.

(1-x)Pb(Mg_1/3_Nb_2/3_)O_3_-xPbTiO_3_ (PMN-PT) is a very important system of relaxor ferroelectrics. 0.67PMN-0.33 PT single crystals show large electromechanical coupling coefficient *k*_33_ (~94%) and piezoelectric constant *d*_33_ (~2800 pC/N)^20^, and very large electromechanical coupling were also observed in a 0.67PMN-0.33 PT film cantilever^21^. In this paper, we focus on the properties of ultrathin 0.67PMN-0.33 PT relaxor ferroelectric films with interest in the following issues. Do the ultrathin PMN-PT films show ferroelectricity? How does the ferroelectric domain form during the cooling process? We fabricated PMN-PT thin films grown on SrRuO_3_ (SRO)/SrTiO_3_ (STO) structure by pulsed laser deposition (PLD) with thicknesses ranging from 10 to 600 nm, and found that with decreasing film thickness the ferroelectric domains change from the multi-domain state to the single domain state with downward self-polarization. We grew 10 nm thick PMN-PT films on 0.7 wt.% Nb-doped SrTiO_3_ (NSTO) substrate and La_0.7_Sr_0.3_MnO_3_ (LSMO)/STO structure, respectively, and found downward self-polarization in PMN-PT/NSTO and upward self-polarization in PMN-PT/LSMO. In order to uncover the self-polarization mechanism, we also fabricated ultrathin PMN-PT films grown on NSTO substrate with thickness ranging from 2 to 30 nm to analyze the variation of the depletion width of PMN-PT/NSTO barrier at different temperatures. We found that downward self-polarization forms in PMN-PT(4 nm)/NSTO sample at room temperature and the temperature of the dielectric response peak for the sample is higher than that for the single crystal. Resistance-voltage measurements and AC impedance analysis for PMN-PT (4 nm)/NSTO sample suggest that Schottky depletion width at high temperatures is smaller than film thickness. We propose that Schottky interfacial dipoles make the dipoles of PNRs in PMN-PT point downward at high temperatures and lead to the self-polarization at room temperature with the assistance of in-plane compressive strain. This work sheds light on the understanding of epitaxial strain effects on relaxor ferroelectric films, as well as self-polarization mechanism.

## Results

**Figure 1:**
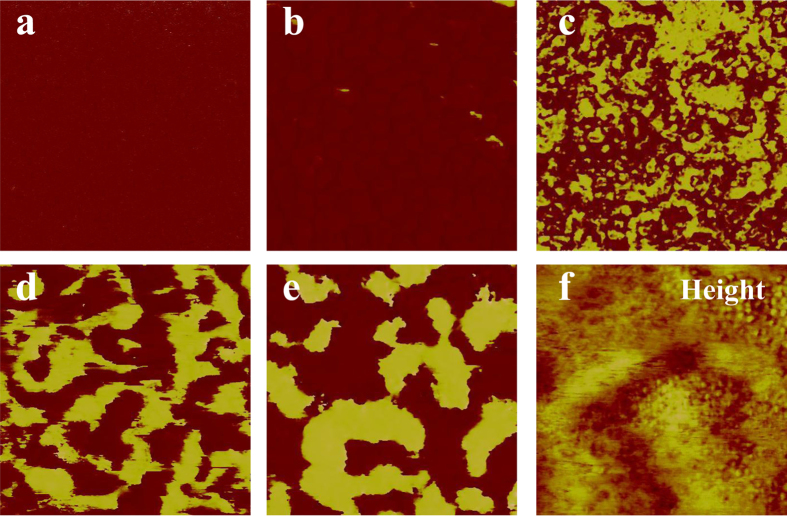
The out-of-plane PFM images, (**a–e**) 50 nm, 100 nm, 200 nm, 400 nm, 600 nm. (**f**) The topographic image for 600 nm. PMN-PT films were deposited on 30 nm thick SRO electrode. All the scan areas are 1 × 1μm^2^.

**Figure 2:**
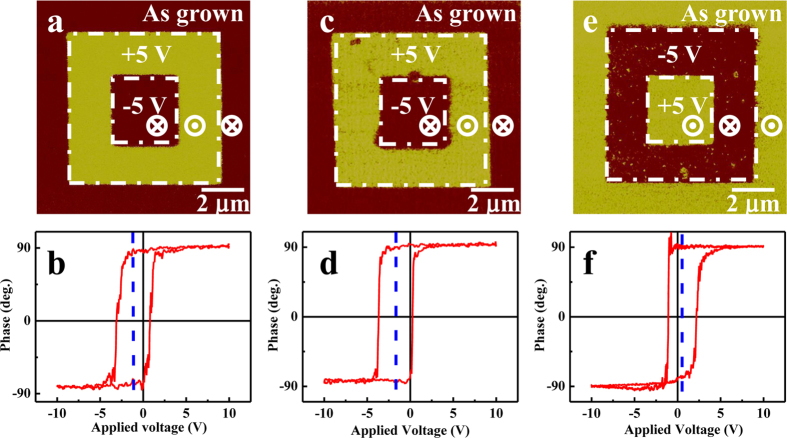
PFM phase images (**a,c,e**) and local PFM hysteresis loops (**b,d,f**) for PMN-PT(10 nm)/NSTO (**a,b**), PMN-PT(10 nm)/SRO(30 nm)/STO (**c,d**) and PMN-PT(10 nm)/LSMO(30 nm)/STO (**e,f**) heterostructures. In (**a,c**) or (**e**), 7 × 7 μm^2^ and 3 × 3 μm^2^ regions were written in the virgin films by applying a dc sample bias of + 5 V (or −5 V) and −5 V (or + 5 V) to the sample, respectively. Vertical blue-dashed line indicates the center of hysteresis loop.

Figure 1a–e shows the out-of-plane piezoresponse force microscopy (PFM) phase images acquired from PMN-PT thin films (50 nm to 600 nm) grown on SRO(30 nm)/STO(001) structures. We found that ferroelectric domains change from the multi-domain state to the single domain state with decreasing film thickness (Later we will see that the dark color represents downward self-polarization). Figure 1f shows the topographic image for the 600 nm thick sample, the root mean square roughness of which is 2.61 nm. We measured PFM phase images and local hysteresis loops on the 10 nm thick PMN-PT films deposited on NSTO, SRO and LSMO electrodes, and the results are shown in Fig. 2. Poling process in Fig. 2a,c was carried out by first applying a dc sample bias of + 5 V to write a 7 × 7 μm^2^ square region and then a dc sample bias of −5 V to write a 3 × 3 μm^2^ square region in the previous positively biased region. Since the original color of the image before poling is the same as that of the negatively poled region, it can be deduced that downward self-polarization forms in the virgin PMN-PT films grown on NSTO and SRO electrodes. By performing the similar procedure, we confirm that the 10 nm thick PMN-PT film grown on LSMO electrode shows an upward self-polarization. The shifts of local PFM hysteresis loops in Fig. 2b,d,f indicate the presence of a biased-voltage due to the different contacts between the bottom (conducting layer) and top (AFM tip) electrodes to PMN-PT. Because the top electrode is the same (AFM tip coped with Pt/Ir) for the three cases, the shifts reflect the induced interface electrostatic potential step due to the built-in electric field at the interface. We analyze energy band diagram of the above three electrodes and obtain the space charges distribution in the interface of PMN-PT/electrode (Supplementary Information S1). The built-in electric fields are upward and downward for PMN-PT/NSTO (or PMN-PT/SRO) and PMN-PT/LSMO, respectively, which lead to the corresponding negative and positive shifts of local PFM hysteresis loops. The XRD patterns for PMN-PT films with different thicknesses can be found in Supplementary Information S2. The variation of lattice parameter c with film thickness suggests huge biaxial epitaxial compression strains in the ultrathin PMN-PT films (Fig. S2c) The c axis parameters for the 10 nm thick PMN-PT films deposited on NSTO, SRO, and LSMO electrodes are 4.100 Ǻ, 4.077 Ǻ and 4.076 Ǻ, respectively (Calculated from Fig. S2d). Compared to PMN-PT crystal lattice parameter (a = 4.019 Ǻ), large in-plane epitaxial compressive strain forms in these ultrathin PMN-PT films. The local hysteresis loops for PMN-PT films with different thicknesses were also measured (Supplementary Information S3). The coercive electric field of ultrathin PMN-PT films calculated from the local hysteresis loops is very large as shown in Fig. S3b. This can be attributed to the in-plane compressive strain that induced the increase of coercive field in ferroelectric films^22^. The in-plane compressive strain provides a large barrier for the switching of polarization.

**Figure 3:**
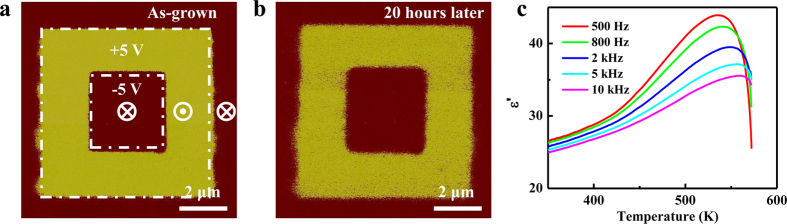
PFM phase images acquired in PMN-PT(4 nm)/NSTO heterostructure just after poling (**a**) and after 20 hours (**b**). 7 × 7 μm^2^ and 3 × 3 μm^2^ regions were written in the virgin films by applying a dc sample bias of + 5 V and −5 V to the sample, respectively. (**c**) Temperature dependence of dielectric permittivity of Au/PMN-PT(4 nm)/NSTO heterostructure measured at different frequencies.

In order to uncover the mechanism of self-polarization, hereafter, we focus on the ultrathin PMN-PT films (2 to 30 nm) deposited on NSTO substrate, especially a 4 nm thick PMN-PT thin film. Figure 3a,b shows the PFM phase images acquired from the 4 nm thick PMN-PT film grown on NSTO just after poling and after about 20 hours. The 4 nm thick PMN-PT film shows downward self-polarization or monodomain, which exists for PMN-PT films with thickness up to 30 nm. For 2 nm thick PMN-PT film, it is difficult to get reliable PFM images because the roughness of the film is comparable to the thickness of the film. Therefore, we demonstrates that ferroelectricity can exist in a 4 nm thick PMN-PT film, which does not support the conclusion of previous report on PbSc_0.5_Nb_0.5_O_3_ and PbMg_1/3_Nb_2/3_O_3_, which suggested that biaxial epitaxial compression favors the relaxor state over ferroelectricity^12^. By comparing Fig. 3a with Fig. 3b, it can be deduced that the two written polarization states are very stable. The temperature dependence of dielectric permittivity of Au/PMN-PT(4 nm)/NSTO heterostructure was measured by AC impedance at various frequencies with a fixed AC signal of 50 mV and the results are shown in Fig. 3c. It can be seen that a peak appears at around 550 K (*T*_m_) with a strong frequency dispersion indicating its relaxor characteristics. This peak is not due to oxygen vacancies (Supplementary Information S4). This peak is related to the transformation that the PNRs in the canonical relaxor become frozen into a nonergodic state^5^. It should be mentioned that the peak temperature is higher than that of the 0.67PMN-0.33PT single crystal (Supplementary Information S4), in contradiction with previous report on PbSc_0.5_Nb_0.5_O_3_ and PbMg_1/3_Nb_2/3_O_3_^12^, which showed that in-plane compressive strain decreases *T*_m_.

In order to understand the ferroelectricity and self-polarization in PMN-PT ultrathin films, we need to study the property of PMN-PT/NSTO in details. It is likely that PMN-PT and NSTO can form a Schottky contact, so current-voltage (*I*-*V*) curve measurements and AC impedance technique are used. As mentioned in Pintilie *et al*.’s papers, the effective charge density in the depleted region (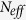) and the Schottky depletion width (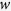) can be estimated by following equations^23^,^24^ (Other parameters are constants or fitted values, and details see Pintilie *et al*.’s papers or Supplementary Information S5):


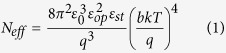



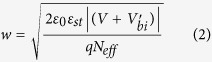


The above equations are valid only if at low voltages the total width of the depletion layers is less than half of the film thickness^24^, so the model may be not suitable for estimating the depletion width in untrathin ferroelectric films at room temperature (Supplementary Information S5). Specially, the depletion width tends to be very small at high temperatures and the model may be valid in ultrathin PMN-PT film. In this paper we demonstrate the direct proof for the change of the Schottky depletion width in 4 nm thick PMN-PT film with temperature increasing experimentally, and estimate the the Schottky depletion width in 4 nm thick PMN-PT film at 500 K by Pintilie *et al*.’s model.

**Figure 4:**
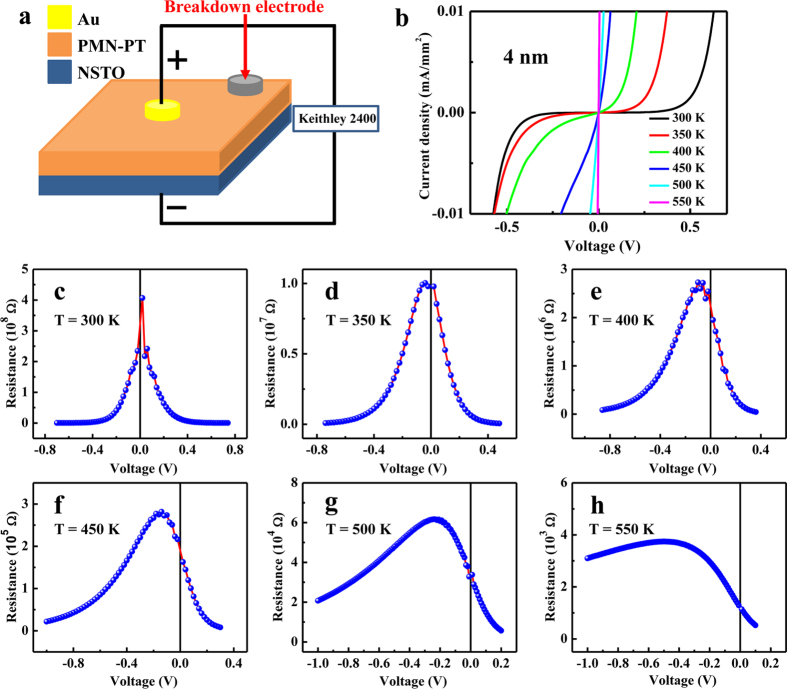
(**a**) Configuration for *I*-*V* measurements. (**b**) *I*-*V* curves of Au/PMN-PT(4 nm)/NSTO heterostructure measured at various temperatures. The sweeping sequence is from positive bias to negative bias. (**c–h**) *R*-*V* curves at various temperatures obtained from (**b**).

Figure 4a shows the measurement configuration. All the measurements were carried out after the electric breakdown between the bottom electrode and top breakdown electrode using silver paste, so ohmic contact was obtained between NSTO substrate and bottom electrode. *I*-*V* curves and complex impedance spectra were measured with top Au electrode as the positive electrode, whose diameter is 500 μm. For electrical transport in heterostructures involving more than one interface, it is important to identify the roles of different interfaces^25^. We confirmed that the resistance in the structure mainly comes from the Schottky interface between PMN-PT films and NSTO substrate, and ferroelectric domain with downward self-polarization is not inversed by the external bias voltage at room temperature in consideration of current leakage (Supplementary Information S6). Figure 4b shows the *I*-*V* curves of Au/PMN-PT(4 nm)/NSTO heterostructure at various temperatures. It can be seen that they show rectifying behavior reflecting the contributions of Schottky contact. The resistance of the heterostructure decreases dramatically with increasing temperature, consistent with the increase of effective charge density with increasing temperature. From Fig. 4b, we can get the resistance-voltage (*R*-*V*) curves as shown in Fig. 4c–h. All these curves show a peak, and with increasing temperature the peak shifts from zero voltage to negative bias voltages and the peak resistance decreases. Since the effective charge density becomes larger with increasing temperature, Schottky depletion width in PMN-PT film becomes smaller with increasing temperature. Because the effective charge density in NSTO is expected to be much larger than that in PMN-PT film, the depletion layer is mainly in PMN-PT film. At 300 K, the peak position is around zero voltage, i.e., the resistance of the heterostructure decreases when negative bias voltages are applied, which suggests that the Schottky depletion width equals to the thickness of PMN-PT film. In contrast, at high temperatures, the peak position shifts to negative bias voltages, which indicates that negative bias voltage makes the depletion width increase until PMN-PT film is completely depleted, resulting in the increase first and then decrease of resistance of the heterostructure. This conclusion can also be drawn by considering the *R-V* curves of PMN-PT films with different thicknesses as shown later. Therefore, the depletion width is smaller than the thickness of PMN-PT film at high temperatures (roughly starting from 400 K). Energy band diagram under external bias voltage in PMN-PT/NSTO interface shows the change of the depletion width with applying external bias voltage on PMN-PT side (Shown in Fig. S14c), and the detailed explanation of the peak in *R*-*V* curves can be found in Supplementary Information S7. Actually, there have been some theoretical work in the literature assuming that the depletion width of ferroelectric thin films is rather thin (a few nanometers)^26^,^27^. In this work, the effective charge density in the depleted region of the 4 nm thick PMN-PT film at 500 K is 6.24 × 10^19^ cm^−3^, and the depletion width is about 0.98 nm (Supplementary Information S5).

**Figure 5:**
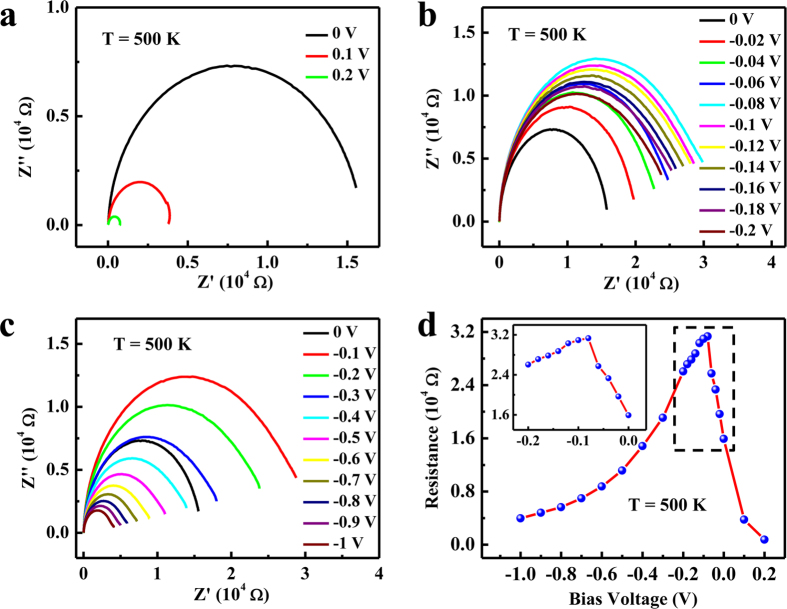
Nyquist plots for sample at 500 K with bias voltage sweeping from 0.2 to 0 V with a 0.1 V step (**a**), from 0 to −0.2 V with a 0.02 V step (**b**) and from 0 to −1 V with a 0.1 V step (**c**), respectively. (**d**) *R*-*V* curves deduced from (**a**) to (**c**). The inset is the expanded view of the dashed square region.

We also used complex impedance spectroscopy^28^ to explore the property of PMN-PT/NSTO heterostructure. Complex impedance spectra under different bias voltages at various temperatures were obtained with Nyquist plots (Supplementary Information S8). Semicircles related with the resistor and capacitor in parallel appear in the Nyquist plots of high temperatures. Figure 5a–c is Nyquist plots for the sample at 500 K with bias voltage sweeping from 0.2 to 0 V with a 0.1 V step, from 0 to −0.2 V with a 0.02 V step and from 0 to −1 V with a 0.1 V step, respectively. From the semicircles in these figures, we got the resistance values under different bias voltages as shown in Fig. 5d. The inset in Fig. 5d is the expanded view of the dashed square region, which shows a resistance peak at around −0.08 V. The behavior of the *R*-*V* curve is similar to the *R*-*V* curve shown in Fig. 4e and suggests that the depletion width of PMN-PT at 500 K is smaller than the thickness of PMN-PT film as mentioned above. Nyquist plots for sample under different negative bias voltages at various temperatures are shown in Fig. S17 and the corresponding *R*-*V* curves for temperature above 450 K are shown in Fig. S18. It can be seen that they show a peak at a certain negative bias voltage, which is consistent with the *R*-*V* curves in Fig. 4f-h.

**Figure 6:**
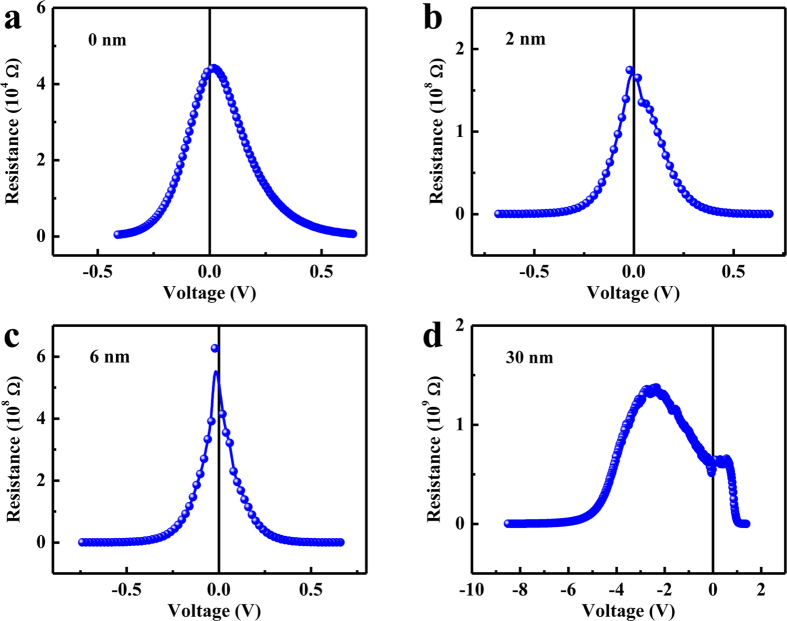
(**a–d**) *R*-*V* curves of Au/PMN-PT/NSTO heterostructures with different thicknesses of PMN-PT measured at room temperature.

As mentioned previously, the depletion width of Schottky contact is smaller than the thickness of PMN-PT film (4 nm) at high temperatures. As a result, the *R*-*V* curves show a peak at a certain negative bias voltage. At room temperature, this behavior should also occur if we grow thicker PMN-PT film to ensure that the depletion width is smaller than the thickness of PMN-PT film. We measured the *I*-*V* curves of Au/PMN-PT/NSTO heterostructure with different thicknesses of PMN-PT at room temperature and the results can be found in Supplementary Information S9 (Fig. S20), from which *R*-*V* curves were obtained as shown in Fig. 6. The sample of 0 nm represents Au/NSTO heterostructure. The resistance peak is around zero voltage for 0, 2, 6 nm thick samples and shifts to negative voltage for 30 nm thick sample, implying that the depletion width is larger than 6 nm and smaller than 30 nm at room temperature. For the 30 nm thick PMN-PT film (Shown in Fig. 6d), Schottky depletion width is smaller than film thickness and negative voltage increases its Schottky depletion width. The current changes very slowly in this process until Schottky depletion width equals to the film thickness, so a peak in *R-V* curve appears at a certain negative bias voltage. If the Schottky depletion width already equals to the thickness of PMN-PT film at zero voltage, applied voltages can not increase Schottky depletion width further. In this case, the resistance drops with increasing negative voltage and the *R-V* curve shows a peak at zero bias voltage. Therefore, the conclusion from the *R*-*V* curves of Au/PMN-PT/NSTO heterostructure with different thicknesses of PMN-PT is consistent with the expectation based on the temperature dependence of *R*-*V* curves for the 4 nm thick sample.

## Discussion

**Figure 7:**
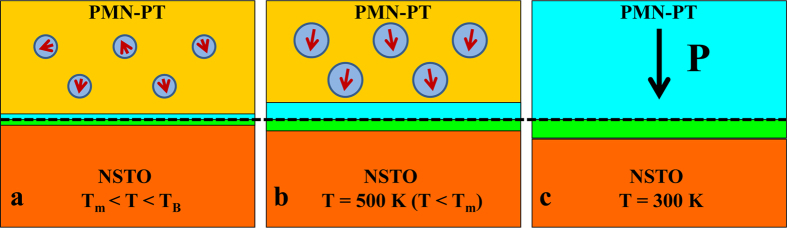
(**a–c**) Schottky interfacial dipole model describing the variation of PNRs and depletion width with decreasing temperatures and self-polarization at room temperature for relaxor ferroelectric PMN-PT thin films with biaxial epitaxial compressive strain. The green and blue regions are the depletion layers in NSTO and PMN-PT, respectively. The circle with a red arrow inside denotes the PNR. The black arrow stands for the self-polarization of PMN-PT. The black dashed line is the interface of PMN-PT/NSTO.

Self-polarization in PMN-PT films is likely induced by the dipoles in PMN-PT/electrode interface, which is determined by the work functions of PMN-PT and the bottom electrode. Since upward self-polarization in PMN-PT/LSMO and downward self-polarization in PMN-PT/SRO were revealed by PFM (Fig. 2), we can deduce that the work function of PMN-PT films is between those of LSMO electrode and SRO electrode. The work function values previously reported for the LSMO film, SRO film, and NSTO substrate are 5.2 ± 0.1, 4.8 ± 0.1, and 4.1 ± 0.1eV^29^. If we difine Ф(material) as the work function of the material, so we can deduce that 4.8 eV < Ф(PMN-PT) < 5.2 eV, and Ф(NSTO) < Ф(PMN-PT). Energy band diagram of PMN-PT/NSTO can be analyzed (Shown in Fig. S14). Considering the aforementioned results and the PNRs in relaxor ferroelectrics, we propose a Schottky interfacial dipole model to account for the ferroelectricity and self-polarization in PMN-PT film grown on NSTO and the schematic illustration of our model is shown in Fig. 7. For PMN-PT/NSTO Schottky contact, a Schottky barrier is formed with negative space charges on PMN-PT side (blue region) and positive space charges on NSTO side (green region) near the interface (the black dashed line), resulting in Schottky interfacial dipoles. As revealed in Fig. 3c, the *T*_m_ of the 4 nm thick PMN-PT is about 550 K and the Burns temperature *T*_B_, at which PNRs start to appear, should be much higher than *T*_m_^7^. When PMN-PT film was cooled down from the deposition temperature (883 K), the sizes of PNRs increase. Moreover, according to the theoretical calculation^17^, biaxial epitaxial compressive strain in relaxor film also increases the sizes of PNRs and aligns the dipoles of PNRs pointing up and down, which is different from the random orientations of dipoles of PNRs in single crystal or films without epitaxial strain. At high temperatures (Fig. 7a,b), the depletion width is smaller than the thickness of PMN-PT film. During the process of cooling down from high temperatures, the Schottky interfacial dipoles interact with the dipoles of PNRs and result in the downward self-polarization (Fig. 7b,c) in PMN-PT. In the meanwhile, the depletion width of PMN-PT increases and equals to the thickness of PMN-PT film at room temperature. With the assistance of in-plane compressive strain, PNRs merge into a single domain state with the self-polarization downward at room temperature. The stability of the written polarization state as shown in Fig. 3b can be understood by considering the biaxial epitaxial compressive strain, which leads to a barrier for polarization switching.

Recently, there are many reports on self-polarization of ultrathin normal ferroelectric films (with thicknesses below 10 nm) just after deposition as revealed by PFM^30^,^31^,^32^,^33^,^34^,^35^,^36^, in addition to some thicker ferroelectric films (with thicknesses above 100 nm)^37^,^38^,^39^,^40^. Several mechanisms have been proposed, such as the Schottky contact related built-in electric field at the ferroelectric/electrode interface^33^,^34^,^41^, surface ionic adsorption^36^, different terminated interface^37^, flexoelectric effects^39^, etc. It is worth pointing out that our Schottky interfacial dipole model is different from the model of Schottky contact related built-in electric field at the ferroelectric/electrode interface^33^,^34^, which assumes that the depletion layer is throughout the entire ferroelectric films and the polarization of ferroelectric films is along the same direction as the built-in electric field. Although our model is proposed for the relaxor ferroelectrics, it should be also suitable for the normal ferroelectrics if the condition of our model is satisfied, i.e., the thickness of depletion (or accumulation) layer for ultrathin ferroelectric film (with a high Curie temperature) is very small at high temperatures and large in-plane compressive strain exists in ferroelectric films at room temperature, so the dipoles of ferroelectric will be aligned by the interfacial dipole of the depletion (or accumulation) layer during the cooling process, resulting in self-polarization with the assistance of in-plane compressive strain.

It should be mentioned that both theoretical work^17^ and experimental work^18^,^19^ suggest that the in-plane compressive strain increases T_m_ of relaxor ferroelectrics, which supports the results of our paper. The only exception is the work by Tyunina *et al*.^12^, which shows that the “in-plane compressive strain decreases T_m_ of PMN”. The in-plane compressive strain for Tyunina *et al*.’s work originates from the lattice mismatch between PMN and La_0.5_Sr_0.5_CoO_3_ (both are thin films), and the thickness of their La_0.5_Sr_0.5_CoO_3_ thin films is 150 nm grown on MgO. This is very different from that of Nagarajan *et al*.’s paper^19^, which has a very thin (40 nm) La_0.5_Sr_0.5_CoO_3_ thin film as electrode and the strain is mainly determined by the lattice mismatch between 0.9PMN-0.1PT film and the substrate. Moreover, for Tyunina *et al*.’s work, La_0.5_Sr_0.5_CoO_3_ thin films got tensile strain from MgO substrate because the in-plane lattice parameter of MgO is larger than that of La_0.5_Sr_0.5_CoO_3_, and the large thickness (150 nm) of their La_0.5_Sr_0.5_CoO_3_ thin films will induce other effect due to the large roughness of thick film besides the strain effect. Therefore, the method for getting the in-plane compressive strain in Tyunina *et al*.’s paper is not straightforward, which makes thing complicated. While in our PMN-PT/SRO/STO, PMN-PT/LSMO/STO and PMN-PT/NSTO heterostructures, because the lattice parameter of PMN-PT has the biggest value in these materials, the formation of in-plane compressive strain in PMN-PT films is clear. To understand the anomalous behavior in Tyunina *et al*.’s paper, a careful characterization of their samples, such as the roughness of the 150 nm thick La_0.5_Sr_0.5_CoO_3_ films and the interface between PMN film and La_0.5_Sr_0.5_CoO_3_ film may be needed. For the Schottky dipole effect, it may be also helpful for the increase of T_m_ besides the in-plane compressive strain. However, Nagarajan *et al*.’s paper^19^ showed that T_m_ approaches the bulk value when the in-plane compressive strain reduces to zero, suggesting that Schottky dipole effect is not the main reason for the increase of T_m_.

The similarity between the relaxor ferroelectrics and the normal ferroelectrics regarding the in-plane compressive strain effect on T_m_ and T_c_ can be understood as follows. The main difference between the relaxor ferroelectrics and the normal ferroelectrics is that PNRs form at Burns temperature and get frozen at T_m_ and becomes ferroelectric at a certain temperature below T_m_. The PNR is nanosized ferroelectric region, and the in-plane compressive strain favors its formation and increases the Burns temperature, the size of PNRs and the resultant increase of T_m_^17^, just as the increase of T_c_ under the in-plane compressive strain for the normal ferroelectrics^13^,^14^,^15^,^16^.

In conclusion, ferroelectricity was observed in the ultrathin relaxor ferroelectric PMN-PT film with large biaxial epitaxial compressive strain. Ultrathin PMN-PT films show upward self-polarization in PMN-PT/LSMO and downward self-polarization in PMN-PT/SRO (or PMN-PT/NSTO), respectively. The written polarization states in ultrathin PMN-PT films are very stable. The peak in the temperature dependence of dielectric permittivity for the 4 nm thick PMN-PT film deposited on NSTO shifts to higher temperatures compared with that of PMN-PT single crystal. *R*-*V* curves and complex impedance spectra suggest that Schottky depletion width is much smaller than the thickness of PMN-PT film at high temperatures. Based on these experimental results, a Schottky interfacial dipole model was proposed to account for the ferroelectricity and self-polarization in the ultrathin relaxor ferroelectric PMN-PT films with the assistance of in-plane compressive strain. This work is helpful for understanding ferroelectricity and self-polarization in the ultrathin relaxor ferroelectric films as well as the ultrathin normal ferroelectric films.

## Methods

Sample preparation: Epitaxial PMN-PT films were deposited on La_0.7_Sr_0.3_MnO_3_ (LSMO), SrRuO_3_ (SRO), and 0.7 wt.% Nb-doped SrTiO_3_ (NSTO) by pulsed laser deposition (PLD) with a KrF excimer laser (*λ* = 248 nm). A target of 0.67PMN-0.33PT with 5% excess of PbO was used in order to compensate the volatility of PbO. The substrate-target distance was fixed at 5 cm, and the pulse energy density was about 1.2 J/cm^2^. During the deposition process, a substrate temperature of 610 °C (720 °C, 700 °C) and an oxygen pressure of 30 (20, 26) Pa for PMN-PT (SRO, LSMO) film were maintained. After PMN-PT film deposition, the film was quenched to room temperature quickly in an oxygen pressure of 0.5 atm in order to suppress the formation of pyrochlore, which is lead-deficient. Au top electrodes were deposited using magnetron sputtering. Ferroelectric domains were measured by using a commercial Scanning Probe Microscope (SPM) system (Multimode 8 SPM, Bruker). Current-Voltage (*I*-*V*) curve measurements at different temperatures were performed using a two-probe method with a Keithley 2400 source meter, and AC impedance measurements at different temperatures were executed by a WK 6500B precision impedance analyzer from 20 Hz to 1 MHz with a fixed AC signal of 50 mV.

## Additional Information

**How to cite this article**: Miao, P. *et al*. Ferroelectricity and Self-Polarization in Ultrathin Relaxor Ferroelectric Films. *Sci. Rep.* **6**, 19965; doi: 10.1038/srep19965 (2016).

## Supplementary Material

Supplementary Information
