# Supplementary material for: Ferroelectricity and Self-Polarization in Ultrathin Relaxor Ferroelectric Films
Source: Sci Rep. 2016 Jan 28;6:19965. doi: 10.1038/srep19965 (PMC4730210; doi:10.1038/srep19965)
Supplement: Supplementary Information [file srep19965-s1.doc]

Supplementary information

Ferroelectricity and Self-Polarization in Ultrathin Relaxor Ferroelectric Films

Peixian Miao^1,2^, Yonggang Zhao^1,2*^, Nengneng Luo^3^, Diyang Zhao^1,2^, Aitian Chen^1,2^, Zhong Sun^1,2^, Meiqi Guo^1,2^, Meihong Zhu^1,2^, Huiyun Zhang^1,2^ and Qiang Li^3^

^1^ Department of Physics and State Key Laboratory of Low-Dimensional Quantum Physics, Tsinghua University, Beijing 100084, China

*^2^ Collaborative Innovation Center of Quantum Matter, Beijing 100084, China*

^3^ Department of Chemistry, Tsinghua University, Beijing 100084, China

Correspondence and requests for materials should be addressed to Y. Z. (ygzhao@tsinghua.edu.cn)

**S1. Monodomain polarization in ultrathin PMN-PT films and space charges distribution in the interface of PMN-PT/electrode**

Since upward self-polarization in PMN-PT/LSMO and downward self-polarization in PMN-PT/SRO were revealed by PFM (Fig. 2), we can deduce that the work function (Ф) of PMN-PT films is between those of LSMO electrode and SRO electrode. The work functions values previously reported for the LSMO film, SRO film, and NSTO substrate are 5.2±0.1, 4.8±0.1, and 4.1±0.1eV^1^. So we can deduce that 4.8 eV < Ф(PMN-PT) < 5.2 eV, and Ф(NSTO) < Ф(PMN-PT). We analyze energy band diagram of above three electrodes in **S7** and obtain the space charges distribution in the interface of PMN-PT/electrode (shown in Fig. S1).

**Figure S1** | (a) and (b) Schematic illustrations of the Schottky contact and self-polarization for the heterostructures at room temperature. The green and blue regions are the depletion or accumulation layers. The black arrow stands for the self-polarization of PMN-PT. The black dashed line denotes the interface of PMN-PT/electrode.

**S2. XRD patterns and local hysteresis loops for PMN-PT films with different thicknesses**

We fabricated several 0.67PMN-0.33PT films on electrode SRO(30 nm)/SrTiO_3_(001) and their XRD patterns are shown in Fig. S2a. We can see that the films are epitaxial well except the 600 nm sample, in which the pyrochlore phase appears. Off-axis phi scans of the rhombohedral non-degenerate 210^R^ reflection as shown in Fig. S2b prove that 400 nm thick PMN-PT film is the rhombohedral phase^2^, which also proves that the films are epitaxial well. Figure S2c shows the variation of lattice parameter c with film thickness, suggesting huge biaxial epitaxial compression strain in the ultrathin PMN-PT films. Figure S2d shows the x-ray diffraction patterns for the 10 nm thick PMN-PT thin films on NSTO(001) substrate, electrode SRO(30 nm)/SrTiO_3_(001) or electrode LSMO(30 nm)/SrTiO_3_(001), confirming that the films are fully c-axis oriented. The c axis parameters for 10 nm thick PMN-PT films deposited on NSTO, SRO, and LSMO electrodes are 4.100 Ǻ, 4.077 Ǻ and 4.076 Ǻ, respectively. Compared to PMN-PT crystal lattice parameter (a = 4.019 Ǻ), large epitaxial compressive strain forms in these ultrathin PMN-PT films.

**Figure S2 |** (a) X-ray diffraction patterns for PMN-PT films with different thicknesses grown on SRO electrode. (b) Off-axis phi scan of rhombohedral non-degenerate 210^R^ reflection of a 400 nm thick PMN-PT film. (c) Thickness dependence of the out-of-plane lattice parameter, which is calculated from (002) peaks (Shown in the inset). (d) X-ray diffraction patterns for 10 nm thick PMN-PT films grown on LSMO, SRO, and NSTO electrodes, respectively.

**S3. Local hysteresis loops for PMN-PT films with different thicknesses**

We used an ac bias of 1000 mV at 80 kHz applied to the sample to measured local hysteresis loops on PMN-PT films mentioned in S2 and the results are shown in Fig. S3a. Local hysteresis loops were measured by the same PFM tip, and the repeatability is very good for each sample as shown in Fig. S4. E_+_ and E- are defined as the positive and negative electric field at zero phase degree in local PFM hysteresis loops, respectively. The coercive electric field (E_c_ = (|E_+_|+|E-|)/2) of ultrathin PMN-PT films calculated from local hysteresis loops is very large as shown in Fig. S3b, which leads to the stable written domains (Shown in Fig. 3a and Fig. 3b). Figure S3b also shows that coercive electric field increases with decreasing film thickness, similar to that reported in the literature^3^, which demonstrated that the large in-plane compressive strain leads to the increase of coercive field in ferroelectric films^3^. The in-plane compressive strain provides a large barrier for the switching of polarization. We conclude that in-plane compressive strain in ultrathin PMN-PT films strengthens the stability of the written ferroelectric domains. Conversely, the written domain in the 600 nm thick PMN-PT film relaxes to multi-domain state very quickly as shown in Fig. S5, which reflects that the ferroelectric domain can be easily inversed, in accordance with small coercive electric field of the sample. We define negative shift in Fig. S3a as built-in electric field (E_built-in_ = (E_+_+E-)/2), which is shown in Fig. S3c. Because of E_+_ > 0 and E- < 0, the absolute value of built-in electric field should be smaller than that of coercive electric field in a local hysteresis loop, so the built-in electric field tends to be zero with increasing film thickness. The built-in electric field induced by the difference of the work function mainly forms in the depleted region near PMN-PT/NSTO interface, and we deduced in the main text that the depletion width is between 6 nm and 30 nm at room temperature. Thus the negative shifts of local PFM hysteresis loops shown in Fig. S3a are more obvious in ultrathin PMN-PT films.

**Figure S3 |** (a) Local PFM hysteresis loops for PMN-PT films with different thicknesses grown on 30 nm thick SRO electrode. (b) The coercive electric fields calculated from (a). (c) The built-in electric fields calculated from (a).

**Figure S4 |** Local PFM hysteresis loops for PMN-PT films with different thicknesses grown on 30 nm thick SRO electrode. These curves, measured by the same PFM tip, show good repeatability.

**Figure S5 |** The out-of-plane PFM images for 600 nm thick PMN-PT film grown on SRO electrode: (a) measured in virgin film, (b) measured just after poling by applying a dc sample bias of -10 V to write a 300 × 300 nm^2^ square region, (c) measured after 20 minutes relaxation from (b). The scan areas are 1×1 μm^2^.

**S4. Temperature dependence of dielectric permittivity and T_m_ for 0.7PMN-0.3PT single crystal, another 4 nm thick PMN-PT film and 4 nm thick LaAlO_3_ film**

We confirmed that the frequency-dependent dielectric peak at around 550 K is due to the relaxor behavior. It should be mentioned, sometimes people took T_m_ as the ferroelectric phase transition temperature T_c_ for PMN-PT because T_c_ is very close to T_m_^4^. As shown in Fig. S6a, the peak related to oxygen vacancies in (001) oriented 0.7PMN-0.3PT single crystal appears at around 700 K, which is consistent with the result of 0.68PMN-0.32PT single crystal in other paper^5^. We measured temperature dependence of dielectric permittivity in an another 4 nm thick PMN-PT film deposited on NSTO substrate, and see a peak before the rapid increase of dielectric permittivity as shown in Fig. S6b. Similar temperature dependence of dielectric permittivity was found for 250 nm thick Pb(Zr_1-x_Ti_x_)O_3_ films^6^. We also fabricated a 4 nm thick LaAlO_3_ film grown on NSTO substrate, and did not find the peak at around 500 K as shown in Fig. S6c. Thus we convince that the peak shown in Fig. 3c is due to the relaxor behavior.

The dielectric permittivity ($\varepsilon'$) of (001) oriented 0.7PMN-0.3PT single crystal was measured as a function of temperature from 800 K to room temperature by AC impedance at various frequencies with a fixed AC signal of 100 mV and the result is shown in Fig. S7. The T_m_ of (1-x)Pb(Mg_1/3_Nb_2/3_)O_3_-xPbTiO_3_ (PMN-PT) crystal can be roughly described by T_m_(°C)=500x-10 (Reference 4), from which we got the T_m_ of the 0.7PMN-0.3PT single crystal to be about 413 K, which is consistent with that shown in Fig. S7. The T_m_ of the 0.67PMN-0.33PT single crystal should be 428 K according to the formula and is much smaller than that of the 4 nm thick PMN-PT film.

**Figure S6 |** Temperature dependence of dielectric permittivity of (a) 0.7PMN-0.3PT single crystal, (b) another 4 nm thick PMN-PT film grown on NSTO substrate and (c) 4 nm thick LaAlO_3_ film grown on NSTO substrate. These ε’-T curves were measured during heating process.

**Figure S7 |** Temperature dependence of dielectric permittivity for 0.7PMN-0.3PT single crystal measured at various frequencies. These ε’-T curves were measured during cooling process.

**S5. The estimation of Schottky depletion width at 500 K in Au/PMN-PT(4 nm)/NSTO heterostructure.**

In order to get Schottky depletion width in ultrathin PMN-PT films near PMN-PT/NSTO interface, we used the analysis mentioned in Pintilie’s paper^7,8^. The model is valid only if at low voltages the total width of the depletion layers is less than half of the film thickness^8^, which is not satisfied for our samples as we demonstrated that the 4 nm thick PMN-PT film is fully depleted at room temperature deduced from the R-V curves. Consistently, the I-V curve measured at room temperature could not be fitted by this model. However, this model is valid for the 4 nm thick PMN-PT film at high temperatures. The analysis using the method mentioned in Pintilie’s paper is as follows.

The formulas are as follows^8^:

$N_{eff}=\frac{8\pi^{2}\varepsilon_{0}^{3}\varepsilon_{op}^{2}\varepsilon_{st}}{q^{3}}(\frac{bkT}{q})^{4}$ (S1)

$w=\sqrt{\frac{2\varepsilon_{0}\varepsilon_{st}|(V+V_{bi}^{'})|}{qN_{eff}}}$ (S2)

$N_{dop}=\frac{2}{q\varepsilon_{0}\varepsilon_{st}[d(1/C^{2})/dV]}$ (S3)

The parameters involved in the above equations are as follows: $N_{eff}$ ─ the effective charge density in the depleted region; $w$ ─ the depletion layer width; $N_{dop}$ ─ “doping” density (hole concentration); $\varepsilon_{0}$ ─ permitivitty of the free space; $\varepsilon_{op}$ ─ optical dielectric constant; $\varepsilon_{st}$ ─ the dielectric constant at low frequency (static); $k$ ─ Boltzmann’ constant; $q$ ─ electron charge; $b$ ─ the slope of the linear fitting of $\ln(|J|)\sim(|V+V_{bi}^{'}|)^{1/4}$ curve; $V_{bi}^{'}$ ─ the apparent built-in potential, which is chosen to obtain the best linear fit on the considered voltage range; C ─ specific capacitance, whose unit is F/m^2^. It should be mentioned that the top and bottom electrodes are identical in Pintilie’s paper, so the sample can be regarded as a back-to-back connection of two identical Schottky contacts. As a result, the I-V curve reflects the reverse bias property of Schottky contact. In our case, the two Schottky contacts are not identical and PMN-PT/NSTO dominates. We fitted the I-V curve in the negative polarity (reverse bias), for which depletion width becomes larger with negative bias voltage increasing. So absolute value for $(V+V_{bi}^{'})$ is considered in formula (S2).

The reported value of refractive index of 0.67PMN-0.33PT films is 2.59 (Ref. 9), and $\varepsilon_{op}$ calculated by the square power of the refractive index is 6.7081. For Au/PMN-PT(4 nm)/NSTO heterostructure, we obtained that the effective charge density ($N_{eff}$) is 6.24×10^19^ cm^-3^ and the Schottky depletion width without an external bias voltage is 0.98 nm at 500 K ($\varepsilon_{op}$= 6.7081, $\varepsilon_{st}$= 53.59, $b$= 4.864, $T$= 500 K, $V_{bi}^{'}$= 0.01 V, as shown in Fig. S8, and the linear fitting parameters are shown in Table S1). The slope fitted from 1/C^2^-V curve in Fig. S19e is -312.10, thus the doping density calculated by formula (S3) is 8.4×10^19^ cm^-3^ at 500 K. The reason for high $N_{eff}$ value is due to high temperature and large doping density.

For the I-V curve measured at room temperature, although it also shows linear fit (Shown in Fig. S9 and Table S2), the effective charge density and Schottky depletion width deduced from the fitting parameters are physically not correct. For example, taking $V_{bi}^{'}$= -1 V ($\varepsilon_{op}$= 6.7081, $\varepsilon_{st}$= 23.43, $b$= 80.83, $T$= 300 K), we can get $N_{eff}$ = 2.70×10^23^ cm^-3^ and $w$= 0.1 nm, which are not correct since the effective charge density at room temperature should be smaller than that at high temperatures and the Schottky depletion width at room temperature should be larger than that at high temperatures. This can be understood since, as mentioned above, this model is valid only for ultrathin ferroelectric films at high temperatures, but not suitable at room temperature.

**Figure S8** | (a) The I-V curve in negative polarity for Au/PMN-PT(4 nm)/NSTO measured at 500 K. (b) Fit curves for different $V_{bi}^{'}$ parameters. (c) Linear fit for best $V_{bi}^{'}$parameter. (d) Frequency dependence of dielectric permittivity ($\varepsilon'$) of Au/PMN-PT(4 nm)/NSTO heterostructure measured by a WK 6500B precision impedance analyzer at 500 K.

**Figure S9** | (a) I-V curve in negative polarity of Au/PMN-PT(4 nm)/NSTO heterostructure measured on Au electrode at 300 K. (b) Fit curves for different $V_{bi}^{'}$parameters. (c) Frequency dependence of dielectric permittivity ($\varepsilon'$) of Au/PMN-PT(4 nm)/NSTO heterostructure measured by a WK 6500B precision impedance analyzer at 300 K.

**S6.** **Dominant contribution of resistance for Au/PMN -PT/NSTO heterostructures**

Figure S10b shows I-V curves for Au/PMN-PT(10 nm)/NSTO, Au/PMN-PT(10 nm)/LSMO(30 nm)/STO and Au/PMN-PT(10 nm)/SRO(30 nm)/STO heterostructures at 300 K. In addition, we measured I-V curves for Au/PMN-PT(30 nm)/NSTO and In/PMN-PT(30 nm)/NSTO heterostructures at 300 K (Fig. S10d). Thus it can be deduced that the resistance in Au/PMN-PT/NSTO heterostructure is mainly from the Schottky interface of PMN-PT/NSTO.

We measured I-V curves on the breakdown electrode and Au electrode, respectively, at 300 K after heating the sample. Figure S11b confirms that the Au electrode is not electric breakdown at high temperatures, and we can see that the current measured on breakdown electrode is much larger than that measured on Au electrode. The I-V curve at 500 K shows rectifying behavior (Shown in Fig. S11c), indicating that the I-V curve is from Schottky effect. We found that the conduction mechanism for our sample can be described by the Newman equation^10^ ($I\sim\exp(\alpha T)\exp(\beta V)$, where α and β are constants, weakly dependent on temperature and voltage), which is suitable for the tunneling process of Schottky contacts or P-N junctions. The I-V curves from Fig. 4b shown in log scale for current (Shown in Fig. S11d) also prove Schottky effect, which is in accordance with Shang’s paper^11^. The rectifying behavior and essentially linear log I-V relations for V > 0 are typical features of the Schottky junctions^11^.

Because the coercive electric field (E_c_) of ultrathin PMN-PT films is very large as shown in Fig. S3b, external bias voltage on Au electrode can not inverse the ferroelectric domain at room temperature in consideration of current leakage (The diameter of Au electrode is 500 μm). Figure S12a and Figure S12b shows the I-V curves for Au/PMN-PT(30 nm)/NSTO and Au/PMN-PT(4 nm)/NSTO heterostructures measured by the process: 0 V → V_+_ → V- → 0 V. The I-V curves overlap indicating that downward ferroelectric domain is not inversed by external bias voltage. In addition, we measured the I-V curve for PMN-PT(4 nm)/NSTO heterostructure by a conductive atomic force microscope (C-AFM) at room temperature (Shown in Fig. S12c), and found I-V curves are separated in forward and backward sweeping of external bias voltage, indicating that ferroelectric domain is inversed by the external bias voltage (The diameter of the tip is only several nanometers). Thus ferroelectric domain in ultrathin PMN-PT films is not inversed by external bias voltage applied on Au electrode at room temperature, and there is no contribution of polarization current in I-V curves measured on Au electrode at room temperature.

**Figure S10 |** (a) Configuration for I-V measurements for Au/PMN-PT(10 nm)/LSMO(30 nm)/STO and Au/PMN-PT(10 nm)/SRO(30 nm)/STO heterostructures. (b) I-V curves for Au/PMN-PT(10 nm)/NSTO, Au/PMN-PT(10 nm)/LSMO(30 nm)/STO and Au/PMN-PT(10 nm)/SRO(30 nm)/STO heterostructures at 300 K. (c) Configuration for I-V measurements for Au/PMN-PT(30 nm)/NSTO and In/PMN-PT(30 nm)/NSTO heterostructures. (d) I-V curves for Au/PMN-PT(30 nm)/NSTO and In/PMN-PT(30 nm)/NSTO heterostructures at 300 K. The sweeping sequence is from positive to negative.

**Figure S11 |** (a) I-V curves measured on breakdown electrode at 300 K before and after heating the sample. (b) I-V curves measured on Au electrode at 300 K after heating the sample. The data of red lines in (a) and (b) are the same. (c) I-V curve measured on Au electrode at 500 K. (d) I-V curves from Fig. 4b shown in log scale for current (V>0).

**Figure S12 |** (a) I-V curves for Au/PMN-PT(30 nm)/NSTO heterostructure measured at 300 K. (b) I-V curves for Au/PMN-PT(4 nm)/NSTO heterostructure measured at 300 K. (c) I-V curves for PMN-PT(4 nm)/NSTO heterostructure measured by a conductive atomic force microscope with sample bias at 300 K. External bias voltage is applied on Au electrode in (a) and (b), and applied on NSTO electrode in (c).

**S7. Energy band diagram of the Schottky contact and the explanation of the peak in R-V curves**

The I-V curve of Au/PMN-PT(30 nm)/NSTO structure shows rectifying behavior reflecting the contributions of Schottky contact (Fig. S12a). In **S6**, we have shown that the rectifying behavior is dominated by the PMN-PT/NSTO interface. Because of Ф(NSTO) < Ф(PMN-PT), the Fermi level of PMN-PT is lower than that of NSTO in energy band diagram before contact, and energy band of PMN-PT bends upward after contact. It can be deduced that PMN-PT film is an p-type semiconductor. If it is a n-type semiconductor, there should be no barrier for electrons (See Fig. S13a), and PMN-PT/NSTO interface forms an ohmic contact, which is not consistent with the rectifying behavior shown in Fig. S12a. Details of the energy band diagram of the Schottky contact are shown in Fig. S13b. It should be mentioned that Pb(Zr, Ti)O_3_ thin film is regarded as a p-type semiconductor due to the volatility of Pb^12^. Similarly, PMN-PT film can also be regarded as a p-type semiconductor.

Energy band diagram of PMN-PT/NSTO films interface (Ф_m_ < Ф_sc_) is shown in Fig. S14. External bias voltages are applied on PMN-PT side. It is worth noting that the depletion width of holes in PMN-PT films increases when a negative bias is applied (V_a_ < 0) and decreases when a positive bias is applied (V_a_ > 0).

Because of Ф(PMN-PT) < Ф(LSMO), the Fermi level of PMN-PT is higher than that of LSMO in energy band diagram before contact, and energy band in PMN-PT bends downward after contact. Thus the accumulation layer of positive charges forms in PMN-PT films near PMN-PT/LSMO interface. On the contratry, the work function of SRO (NSTO) electrode is lower than that of PMN-PT, and the depletion layer of holes forms in PMN-PT films near PMN-PT/SRO (NSTO) interface, i.e., negetive space charges locate in the depletion region. The work function of Au electrode is 5.1 eV^13^. So, the work-function difference between PMN-PT and Au is quite small, in contrast to the large difference between PMN-PT and NSTO, suggesting the dominant role of PMN-PT/NSTO in determining the resistance for Au/PMN-PT/NSTO heterostructures.

When the PMN-PT film is thick (30 nm), Schottky depletion width in PMN-PT film is smaller than film thickness at room temperature, and negative voltage increases its Schottky depletion width in thicker PMN-PT film. As a result, the current increases very slowly and the resistance increases for negative voltages below 2.5 V, as shown in Fig. S15b and Fig. S15c. For negative voltages above 2.5 V, Schottky depletion width equals to the film thickness (Our films are not too thick), and the resistance should remain constant. However, the electric fields for negative voltages above 2.5 V are roughly larger than 10^6^ V/cm, which are strong fields and can cause the resistance drop of the Schottky contact due to leakage or soft breakdown^14,15^. Actually, leakage current is an important problem for ferroelectric films^14^. This leads to the remarkable increase of current (Fig. S15b) and the resistance peak in the R-V curve (Fig. S15c). For ultrathin ferroelectric film (4 nm thick), Schottky depletion width in PMN-PT film may equal to the film thickness at room temperature. If the Schottky depletion width already equals to the thickness of PMN-PT film at zero voltage, applied negative voltages can not increase Schottky depletion width further. In this case, the current increases quickly and the resistance drops with increasing negative voltage due to the strong electric field related to the ultrathin ferroelectric film. As a result, the R-V curve shows a peak at around zero voltage. It should be mentioned that ferroelectric domain with downward self-polarization is not inversed by applying the external bias voltage on Au electrode at room temperature (Shown in Fig. S12).

**Figure S13 |** Energy band diagrams (Ф_m_ < Ф_sc_). (a) Ohmic contact for n-type semiconductor. (b) Schottky barrier for p-type semiconductor.

**Figure S14 |** Energy band diagram of PMN-PT/NSTO (Ф_m_ < Ф_sc_). (a) Before contact. (b) After contact. (c) Under external bias voltage (V_a_) on PMN-PT film. The depletion width in PMN-PT film increases when a negative bias voltage is applied (V_a_ < 0) on PMN-PT side, and decreases when a positive bias voltage is applied (V_a_ > 0) on PMN-PT side.

**Figure S15 |** (a) I-V curve for Au/PMN-PT(30 nm)/NSTO heterostructure at 300 K. The inset is the expanded view at -2.5 V. (b) $\log(|I|)\sim\log(|V|)$ curve for Au/PMN-PT(30 nm)/NSTO structure in negative voltage range at 300 K . (c) R-V curve calculated from (a). The peak of R-V curve appears at -2.5 V.

**S8. Nyquist plots under different bias voltages at various temperatures**

Figure S16 shows Nyquist plots under zero bias voltage at various temperatures. Semicircle, by which we can obtain the resistance of Schottky interface, appears in Fig. S16 above 450 K. Figure S17 shows Nyquist plots under different negative bias voltages at various temperatures. The radius of semicircle increases first and then decreases with the increase of negative bias voltage above 450 K, which is consistent with the R-V curves in Fig. 4. Fitted R-V curves above 450 K are plotted in Fig. S18. We calculated the capacitance of the sample from semicircles shown in Fig. S17 (Formula: ωRC=1, Reference 16), and 1/C^2^-V curves are shown in Fig. S19. All of these 1/C^2^-V curves show a linear behavior, which are typical features of the Schottky contact and p-n junctions^17^. It should be mentioned that semicircles can not be obtained for the Nyquist plots with low voltages measured close to room temperature, so capacitance can be deduced only for voltages above a certain value. The linear behavior 1/C^2^-V curves measured close to room temperature suggests that NSTO also contributes to the depletion width for these voltages. It should also be pointed out that capacitance of the sample increases with increasing temperature (See Fig. S19), consistent with the decrease of depletion width with increasing temperature.

**Figure S16 |** Nyquist plots for Au/PMN-PT(4 nm)/NSTO heterostructure measured at various temperatures. The inset is the expanded version of the black dotted box.

**Figure S17 |** (a)-(f) Nyquist plots of the sample under different negative bias voltages at various temperatures.

**Figure S18 |** (a)-(c) R-V curves calculated from Nyquist plots at 450 K, 500 K and 550 K.

**Figure S19** | (a)-(f) 1/C^2^-V curves calculated from Fig. S17. Only semicircles can be used to calculate capacitance, so the voltage ranges at different temperatures are not the same.

**S9.** **I-V curves for Au/PMN-PT/NSTO with different thicknesses of PMN-PT at room temperature**

In order to prove that the Schottky depletion width of PMN-PT is very small, the I-V curves for Au/PMN-PT/NSTO with different thicknesses of PMN-PT films were measured at room temperature and the results are shown in Fig. S20. Figure S20b is the expanded version of the black dotted box in Fig. S20a. It can be seen that the negative threshold voltage becomes larger with increasing film thickness. The sample of 0 nm represents Au/NSTO heterostructure.

**Figure S20 |** (a) I-V curves for Au/PMN-PT/NSTO with different thicknesses of PMN-PT films measured at room temperature with the sweeping sequence from positive to negative. (b) Expanded version of the black dotted box in (a).

**References**

1 Minohara, M. *et al.* Band diagrams of spin tunneling junctions La_0.6_Sr_0.4_MnO_3_ /Nb:SrTiO_3_ and SrRuO_3_/Nb:SrTiO_3_ determined by *in situ* photoemission spectroscopy. *Appl. Phys. Lett.* **90**, 132123 (2007).

2 Zavaliche, F. *et al.* Multiferroic BiFeO_3_ films : domain structure and polarization dynamics. *Phase Transition* **79**, 991 (2006).

3 Pertsev, N. A. *et al.* Coervice field of ultrathin Pb(Zr_0.52_Ti_0.48_)O_3_ epitaxial films. *Appl. Phys. Lett.* **83**, 3356 (2003).

4 Feng, Z. *et al.* Dependence of high electric-field-induced strain on the composition and orientation of Pb(Mg_1/3_Nb_2/3_)O_3_-PbTiO_3_ crystals. *Solid State Commun.* **126**, 347-351 (2003).

5 Wang, C. *et al.* High-Temperature Dielectric Relaxation in Pb(Mg_1/3_Nb_2/3_)O_3_–PbTiO_3_ Single Crystals. *J. Am. Ceram. Soc*. **96***,* 1521-1525 (2013).

6 Sheen, D. *et al.* Dielectric and polarization switching anomalies near the morphotropic phase boundary in Pb(Zr_1-x_Ti_x_)O_3_ ferroelectric thin films. *Phys. Rev. B* **67***,* 144102 (2003).

7 Pintilie, L., Alexe, L. Metal-ferroelectric-metal heterostructures with Schottky contacts. I. Influence of the ferroelectric properties. *J. Appl. Phys.* **98**, 124103 (2005).

8 Pintilie, L. *et al.* Metal-Ferroelectric-metal structures with Schottky contacts. II. Analysis of the experimental current-voltage and capacitance-voltage characteristics of Pb(Zr, Ti)O_3_ thin films. *J. Appl. Phys.* **98**, 124104 (2005).

9 Shinozaki, K. *et al.* Fabrication and Optical Properties of Pb(Mg_1/3_Nb_2/3_)O_3_-PbTiO_3_ Thin Films on Si Substrates Using the PLD Method. *IEEE Trans. Ultrason. Ferroelectr. Freq. Control* **55**, 1023 (2008).

10 Sharma, B. L., Purohit, R. K. Semiconductor Heterojunctions. 1-13 (Pergamon Press, Oxford, 1974).

11 Shang, D. S. *et al.* Electronic transport and colossal electroresistance in SrTiO_3_ : Nb-based Schottky junction. *Appl. Phys. Lett.* **94**, 052105 (2009).

12 Lee, J. *et al.* Built-in voltages and asymmetric polarization switching in Pb(Zr,Ti)O_3_ thin film capacitors. *Appl. Phys. Lett.* **72**, 3380 (1998).

13 Michaelson, H. B. The work function of the elements and its periodicity. *J. Appl. Phys.* **48**, 4729 (1977).

14 Nagaraj, B. *et al.* Leakage current mechanisms in lead-based thin-film ferroelectric capacitors. *Phys. Rev. B* **59***,* 16022 (1999)

15 Selders, J. *et al.* Schottky-Barriers on p-Type GalnAs. *IEEE Trans. Electron. Devices* **32**, 605 (1985)

16 Irvine, J. T. S., Sinclair, D. C. & West, A. R. Electroceramics: Characterization by Impedance Spectroscopy. *Adv. Mater.* **2**, 132 (1990).

17 Sze, S. M., Ng, K. K. Physics of Semiconductor Devices. 138-139 (Wiley, New York, 2007).
